# Prognostic value of native T1 and extracellular volume in patients with immunoglubin light-chain amyloidosis

**DOI:** 10.1186/s12872-024-03756-8

**Published:** 2024-02-16

**Authors:** Yumeng Liu, Lingjie Wang, Jingfen Zhu, Meng Chen, Mo Zhu, Yingyu Dai, Chunhong Hu

**Affiliations:** Department of Radiology, The First Affiliated Hospital of Soochow University, Suzhou, 215006 China

**Keywords:** Immunoglubin light-chain amyloidosis, Cardiac magnetic resonance, Cardiac amyloidosis, Native T1, Extracellular volume

## Abstract

**Background:**

Cardiac involvement in patients with immunoglubin light-chain amyloidosis (AL) is a major determinant of treatment choice and prognosis, and early identification of high-risk patients can initiate intensive treatment strategies to achieve better survival. This study aimed to investigate the prognostic value of native T1 and ECV in patients with AL-cardiac amyloidosis (CA).

**Methods:**

A total of 38 patients (mean age 59 ± 11 years) with AL diagnosed histopathologically from July 2017 to October 2021 were collected consecutively. All patients were performed 3.0-T cardiac magnetic resonance (CMR) including cine, T1 mapping, and late gadolinium enhancement (LGE). Pre- and post-contrast T1 mapping images were transferred to a dedicated research software package (CVI42 v5.11.3) to create parametric T1 and ECV values. In addition, clinical and laboratory data of all patients were collected, and patients or their family members were regularly followed up by telephone every 3 months. The starting point of follow-up was the time of definitive pathological diagnosis, and the main endpoint was all-cause death. Kaplan-Meier analysis and Cox proportional risk model were used to evaluate the association between native T1 and ECV and death in patients with CA.

**Results:**

After a median follow-up of 27 (16, 37) months, 12 patients with CA died. Kaplan-Meier analysis showed that elevated native T1 and ECV were closely associated with poor prognosis in patients with CA. The survival rate of patients with ECV > 44% and native T1 > 1389ms were significantly lower than that of patients with ECV ≤ 44% and native T1 ≤ 1389ms (Log-rank *P* < 0.001), and was not associated with the presence of LGE. After adjusting for clinical risk factors and CMR measurements in a stepwise multivariate Cox regression model, ECV [risk ratio (HR):1.37, 95%CI: 1.09–1.73, *P* = 0.008] and native T1 (HR:1.01, 95%CI: 1.00-1.02, *P* = 0.037) remained independent predictors of all-cause mortality in patients with CA.

**Conclusions:**

Both native T1 and ECV were independently prognostic for mortality in patients with CA, and can be used as important indicators for clinical prognosis assessment of AL.

## Introduction

Immunoglobulin light-chain amyloidosis (AL) is the most common systemic amyloidosis results from the deposition of protein derived from fragments of clonal immunoglobulin light chain by abnormally functioning plasma cells. Cardiac involvement is described in up to 70% of AL patients during the course of the disease. Previous studies have shown that cardiac damage is the most important determinant of prognosis in AL patients [1, 2]. In these patients, aggressive chemotherapy and autologous stem cell transplantation (ASCT) can significantly improve the clinical prognosis [3, 4]. Therefore, early recognition of cardiac amyloidosis (CA) may potentially alter prognosis by prompting early aggressive treatment.

​ In current clinical practice, endomyocardial biopsy (EMB) is considered as the “gold standard” for the diagnosis of cardiac amyloidosis, however, the invasive methodology and complicate sampling processing and assessment are the major disadvantages for EMB [5]. Moreover, some non-invasive techniques, such as cardiac biomarkers, electrocardiograph (ECG), and echocardiography can assist physicians with the diagnosis of CA. In previous study, echocardiography was frequently used to diagnostic and prognosticate CA [2, 6], but coexisting causes of left ventricular hypertrophy may affect explanation. Low limb lead voltages, or fragmented QRS complexes on the ECG was also can be used to predict CA [7, 8], but was puzzled by conduction abnormalities and pericardial effusions. Nevertheless, current prognostic predictors of CA rely on surrogate measurements rather than direct markers of interstitial expansion.

In recent years, cardiac magnetic resonance examination (CMR) has emerged as a non-invasive technique for CA diagnosis, because it can provide unique information about tissue composition with late gadolinium enhancement (LGE), which shows a typical pattern of diffuse subendocardial or transmural enhancement rarely seen in other cardiomyopathies [9]. Moreover, Native T1 representing the intrinsic signal of the myocardium and myocardial extracellular volume (ECV) can quantitatively measure the progression of cardiac amyloid infiltration from early infiltration to diffuse transmural involvement [10, 11]. As our previous study has reported [12], native T1 and ECV have good diagnostic accuracy in the diagnosis of CA, especially could detect early disease, and have been shown to correlate with disease burden. Here, the aim of this study was to investigate the prognostic value of native T1 and ECV in patients with AL-cardiac amyloidosis.

## Methods

### Participants

38 AL patients were consecutively recruited from Department of Radiology, the First Affiliated Hospital of Soochow University from July 2017 to October 2021. All these patients were confirmed with systemic AL by Congo red and immunohistochemical staining using specimens of subcutaneous abdominal fat (*n* = 20), bone marrow (*n* = 10), kidney (*n* = 7), and upper gastrointestinal tract (*n* = 1). The diagnostic criteria for cardiac involvement in AL patients [13]: echocardiography showed a ventricular septal thickness of > 12 mm (without other causes) or NT-proBNP > 332ng/L (without renal insufficiency).

### Clinical data

Clinical and laboratory data were collected from all patients, including: gender, age, clinical symptoms and signs, organs involved, ECG manifestations, cardiac biomarkers (NT-proBNP and Troponin T), serum free light chain (sFLC), the difference between involved and uninvolved sFLC (dFLC), Mayo stage and treatment methods, etc. A blood sample was obtained from all these subjects for biochemical examination and hematocrit at about 30 min before the CMR scan. The study was approved by the Ethics Committee of the First Affiliated Hospital of Soochow University (No: 2,019,112) and written informed consent was obtained from all participants.

### CMR protocol

All participants underwent a standard CMR examination with a 3.0-T clinical scanner (Magnetom Skyra, Siemens AG, Healthcare Sector, Erlangen, Germany). A balance steady-state free precession (SSFP) sequence was used to obtain cine images, including multiple slice short-axis images, 2-chamber and 4-chamber long-axis images. The cine image parameters were as follows: repetition time (TR) 39.2 ms, echo time (TE) 1.4 ms, slice thickness 8 mm, field of view (FOV) 174 × 208 mm, matrix 256 × 256. LGE images was performed 10 min after a cumulative dose of 0.1 mmol/kg of gadolinium-based contrast agent (Magnevist, Bayer Healthcare, Berlin, Germany), using the phase-sensitive inversion recovery (PSIR) sequence for acquirement of the same multiple short-axis and long-axis images as cine images. Parameters for LGE images were TR 700 ms, TE 1.5ms, slice thickness 8 mm, flip angle 20º, FOV 256 × 192 mm, matrix 256 × 256, inversion time 300 ms. Native and post-contrast T1 mapping images, including basal, mid-ventricular and apical short-axis images were acquired using the shortened modified look-locker inversion recovery sequence (shMOLLI) before and 15 min after administration of contrast agent. Parameters for T1 mapping study were TR 277.9 ms, TE 1.1 ms, slice thickness 8 mm, flip angle 35 º, FOV 256 × 192 mm, matrix 192 × 144.

### CMR image analysis

The cine images were transferred to a dedicated research software (CVI42 v5.11.3, Circle Cardiovascular Imaging, Alberta, Canada) to calculate left ventricular ejection fraction (LVEF), left ventricular end-diastolic volume index (LVEDVi), left ventricular end-systolic volume index (LVESVi), left ventricular myocardial mass index (LVMI), left ventricular output per wave (LVSV) and cardiac output (CO). Pre- and post-contrast shMOLLI sequence generated images with varying inversion times were transferred to software to create parametric T1 and ECV pixel maps and corresponding values. The LGE images were visually analyzed for the presence or absence of enhancement.

### Follow-up and outcome

The primary outcome for this study was all-cause mortality. Follow-up information was obtained by telephone interviews of the patient or his or her family every 3 months. Patients were followed up until 31 October 2021, or censored if they were alive.

### Statistical analysis

SPSS Statistics Version.22.0 (IBM, Chicago, IL, USA) was used for statistical analysis. Continuous data were expressed as mean ± SD or median and interquartile range according to whether normal distribution or not. Categorical data were expressed as frequencies and percentages. The independent sample t-test or non-parametric Mann-Whitney U test was used to compare the measurement data according to whether they were in accordance with normal distribution. Group count data were analyzed by χ^2^ test or exact probability method. Kaplan-Meier method was used for survival analysis, and log-rank was used to compare the differences between groups. Cox proportional hazard model was used for univariate and multivariate analysis. Statistical significance was defined as *P* < 0.05.

## Results

### Clinical and biochemical markers

The mean age of 38 patients was 59 years (range, 29–78 years), 74% were men. The clinical manifestations, underlying diseases, other organ involvement and ECG findings are shown in Table 1. In this study, according to 2012 clinical staging standard of Mayo Clinic [14], there were 8 (21%), 11 (29%), 10 (26%) and 9 (24%) patients in Mayo stage I, II, III and IV, respectively. Among them, 23 patients received chemotherapy or ASCT, and the remaining 15 patients received expectant treatment (Table 2).

**Table 1:** Clinical data of all patients

Clincal data	All patients(*n* = 38)
**Clinical manifestation**	
Chest tightness and shortness of breath	15(39%)
Fatigue	11(29%)
Edema of both lower limbs	11(29%)
Proteinuria	8(21%)
Dropsy of serous cavity	3(8%)
Syncope	3(8%)
Abnormality of globulin	3(8%)
Back pain	2(5%)
Numbness at the extremities	2(5%)
Arrhythmia	1(3%)
Postural hypotension	1(3%)
Skin change	1(3%)
Hypertrophy of tongue	1(3%)
**Underlying disease**	
Hypertension	13(34%)
Diabetes	3(8%)
Cerebral infarction	5(13%)
Hepatitis B	1(3%)
Bronchial asthma	1(3%)
Ankylosing spondylitis	1(3%)
**Other organ involvement**	
Kidney	15(39%)
Peripheral nerve	5(13%)
Skin	1(3%)
Tongue	1(3%)
Stomach	1(3%)
**Electrocardiogram manifestation**	
Limb lead low voltage	8(21%)
Pseudo infarct presence	13(34%)
Right axis deviation	5(13%)
Atrial and ventricular premature beats	4(11%)
Abnormal ST-T	12(32%)
Right bundle branch block	1(3%)
Left bundle branch block	2(5%)
Atrioventricular block	3(8%)

**Table 2:** Clinical and CMR parameters of patients with ECV ≤ 44% or ECV>44%

Clinical and CMR parameters	All patients (*n* = 38)	Patients with ECV ≤ 44% (*n* = 22)	Patients with ECV>44% (*n* = 16)	*P* value
Male(%)	28(74%)	15(68%)	13(81%)	0.669
Age(years)	59 ± 11	56 ± 13	63 ± 6	0.172
NT-proBNP(pg/mL)	950(418–3541)	614(175–1354)	3879(1245–11,415)	0.003
Troponin T(ng/mL)	0.034(0.014–0.053)	0.024(0.012–0.037)	0.050(0.035–0.060)	0.008
dFLC (mg/L)	167(93–272)	149(51–192)	290(129–969)	0.019
Mayo stage				
I	8(21%)	7(32%)	1(6%)	0.166
II	11(29%)	9(41%)	2(13%)	0.166
III	10(26%)	5(23%)	5(31%)	0.843
IV	9(24%)	1(4%)	8(50%)	<0.001
Native T1 (ms)	1399 ± 103	1342 ± 82	1479 ± 71	<0.001
LVEDVi (mL/m^2^)	72 ± 13	73 ± 15	71 ± 8	0.934
LVESVi (mL/m^2^)	30 ± 3	29 ± 3	31 ± 3	0.402
LVEF (%)	54 ± 9	58 ± 7	49 ± 8	0.007
LVMI (g/m^2^)	117 ± 23	107 ± 23	132 ± 14	0.004
LVSV (mL)	66 ± 13	73 ± 10	56 ± 11	<0.001
Therapeutic method				
Chemotherapy or ASCT	23(61%)	16(73%)	7(44%)	0.201
Expectant treatment	15(39%)	6(27%)	9(56%)	0.201

Data was expressed as mean ± standard deviation (SD) or median (interquartile range). NT-proBNP: N-terminal pro-B-type natriuretic peptide; dFLC: the difference between involved and uninvolved serum free light chain; LVEDVi: left ventricular end-diastolic volume index; LVESVi: left ventricular end-systolic volume index; LVEF: left ventricular ejection fraction; LVSV: left ventricular stroke volume; ASCT: autologous stem cell transplantation

### CMR structural and functional parameters

According to the median ECV (44%) as the critical reference value, patients with CA were divided into ECV ≤ 44% group and ECV > 44% group. As shown in Table 2, NT-proBNP, Troponin T, dFLC, native T1 and LVMI in ECV > 44% group were higher than those in ECV ≤ 44% group, while LVEF and LVSV were lower than ECV ≤ 44% group (*P* < 0.05).

### Survival analysis

**Fig. 1:**
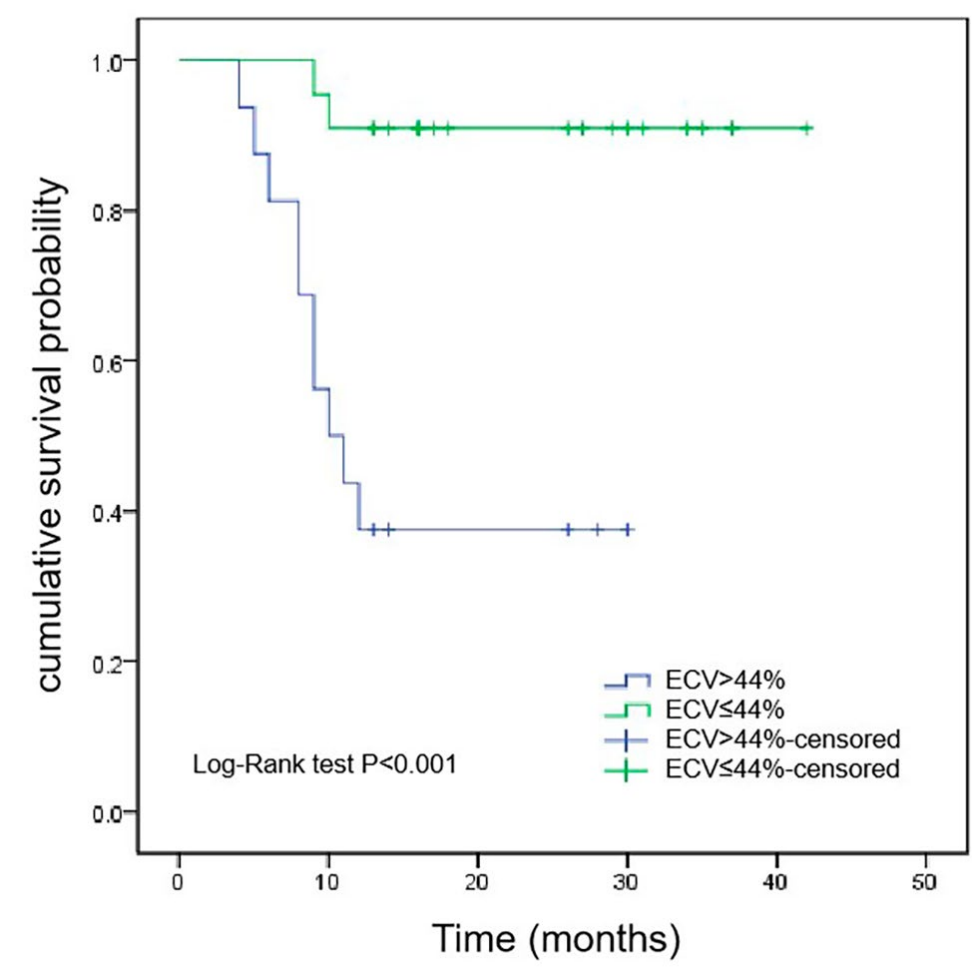
Kaplan-Meier survival curves for patients with ECV > 44% and ECV ≤ 44%

**Fig. 2:**
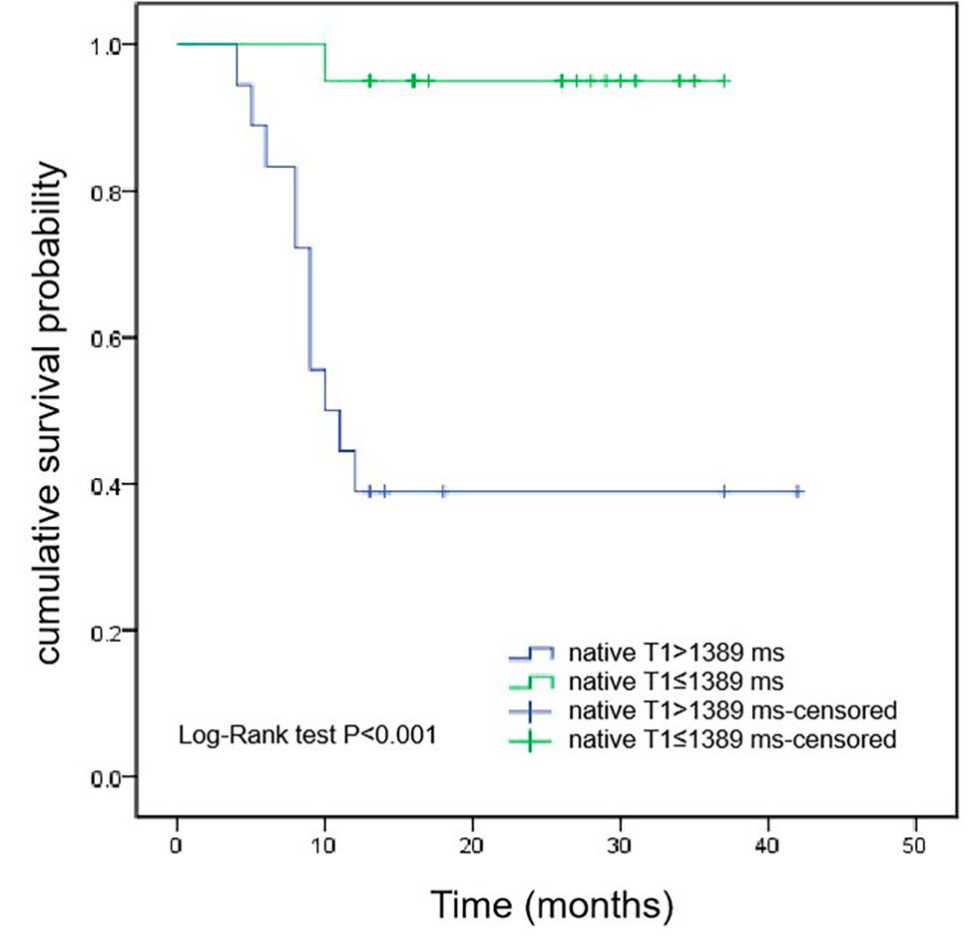
Kaplan-Meier survival curves for patients with native T1 > 1389ms and native T1 ≤ 1389ms

**Fig. 3:**
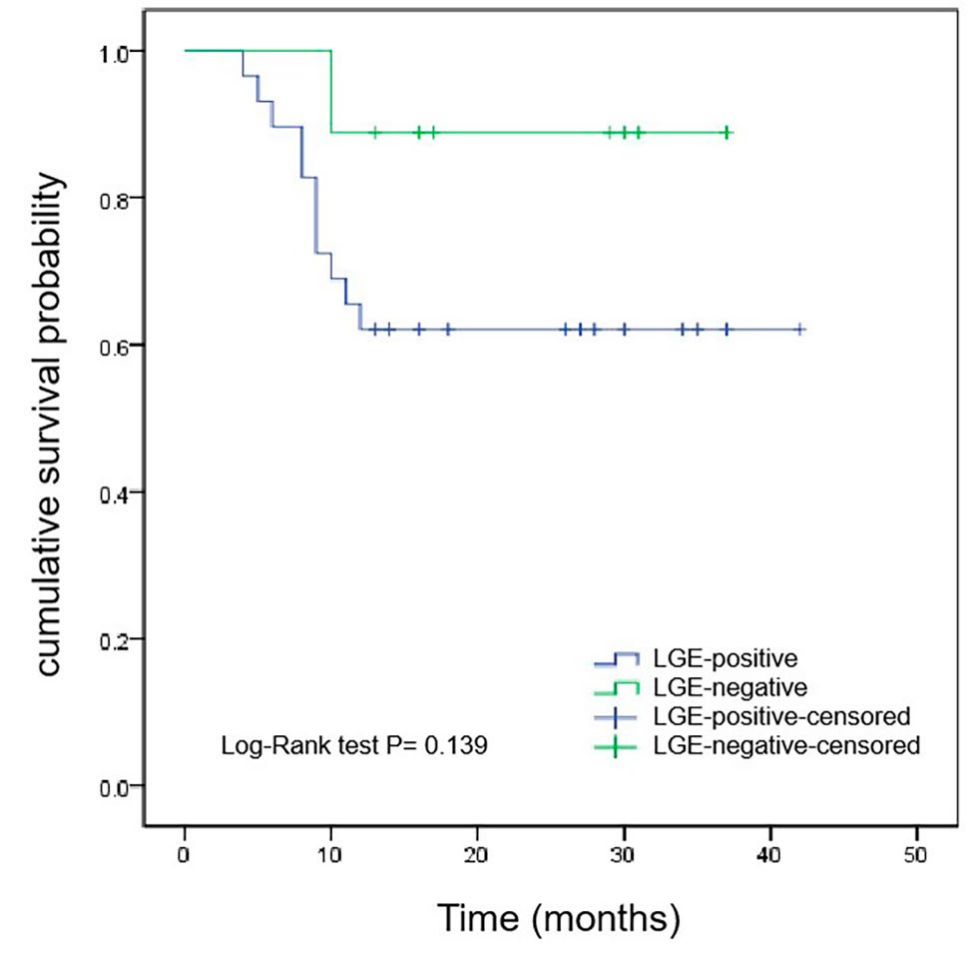
Kaplan-Meier survival curves for patients with LGE-positive and LGE-negative

After a median follow-up of 27 (16, 37) months, 12 patients with CA died. Kaplan-Meier survival curves demonstrated that the survival time of ECV > 44% group was shorter than that of ECV ≤ 44% group (median survival time, 10 months vs. 22 months, *P* < 0.001) (Fig. 1). According to the median native T1 (1389ms) as the critical reference value, patients were divided into native T1 > 1389ms group and native T1 ≤ 1389ms group. As shown in Fig. 2, the survival time of native T1 > 1389ms group was shorter than that of native T1 ≤ 1389ms group (median survival time, 10.5 months vs. 26.5 months, *P* < 0.001). However, Kaplan-Meier survival curves showed that there was no significant difference in the survival time of patients between LGE-positive group and LGE-negative group (*P* = 0.139) (Fig. 3).

In the univariate Cox regression analysis (Table 3), NT-ProBNP, Troponin T, Mayo stage III to IV, native T1, ECV and LVMI were demonstrated as univariate predictors. After adjusting for clinical risk factors and CMR measurements in a stepwise multivariate Cox regression model, only ECV [Hazard ratio (HR): 1.37, 95%CI: 1.09–1.73, *P* = 0.008] and native T1 (HR: 1.01, 95%CI: 1.00-1.02, *P* = 0.037) were independent predictors for the primary endpoint (Table 3).

**Table 3:** Cox proportion hazards ratio models for overall survival

Variable	Univariate Analysis			Multivariate Analysis	
	HR(95%CI)	*P* Value		HR(95%CI)	*P* Value
Age(>60 year)	3.205(0.867–11.848)	0.081			
Gender	1.435(0.431–4.775)	0.556			
NT-proBNP	1.000(1.000–1.000)	<0.001			
Troponin T	1.018(1.002–1.036)	0.032			
Mayo stage III to IV	7.264(1.586–33.267)	0.011			
Native T1	1.015(1.008–1.022)	<0.001		1.010(1.001–1.019)	0.037
ECV	1.397(1.137–1.717)	0.001		1.370(1.085–1.731)	0.008
LGE-positive	4.085(0.527–31.669)	0.178			
LVEDVi	1.018(0.980–1.058)	0.356			
LVESVi	1.180(0.965–1.442)	0.106			
LVEF	0.914(0.873–0.958)	<0.001			
LVMI	1.027(1.001–1.053)	0.038			
LVSV	0.921(0.878–0.967)	0.001			
Chemotherapy or ASCT	0.267(0.080–0.888)	0.031			

## Discussion

Cardiac involvement is the main determinant of treatment choice and prognosis in patients with AL. Early identification of high-risk patients can begin to strengthen treatment strategies to achieve a better survival rate [15, 16]. Cardiac serum biomarkers NT-proBNP and troponin T are commonly used to evaluate the prognosis of patients with CA. Palladini G et al. [17] reported that NT-proBNP was a sensitive marker for cardiac involvement and a prognostic marker for patients with AL. Dispenzieri A et al. [18] found that the increase of troponin T level in patients with CA was related to low survival rate. Mayo clinic staging based on these biomarkers has become an important clinical tool for risk stratification of AL patients [14, 19]. In this study, in the univariate Cox proportional hazard model, NT-ProBNP (HR = 1.00, 95%CI: 1.00–1.00, *P* < 0.001), troponin T (HR = 1.02, 95%CI: 1.00-1.04, *P* = 0.032) and Mayo stage III to IV (HR = 7.26, 95%CI: 1.59–33.27, *P* = 0.011) were risk factors affecting the survival of patients with CA.

In recent years, noninvasive imaging based on cardiac morphology and function has attracted extensive attention in the prognostic assessment of CA [9, 10, 20, 21]. CMR-LGE is not only a diagnostic marker for patients with CA, but also a powerful predictor of mortality [4, 22, 23]. Fontana M et al. [24] reported that patients with CA without LGE had the best prognosis, while those with diffuse transmural LGE had the worst prognosis. In our previous study, we found that nativeT1 and ECV were more sensitive than LGE in the early diagnosis of CA, and the degree of amyloid infiltration in the myocardium could be indirectly reflected by nativeT1 and ECV [12]. In this study, we used nativeT1 and ECV to evaluate the prognostic of patients with CA. The results showed that the increase of nativeT1 and ECV were closely related to the poor prognosis of patients. The survival rate of patients in ECV > 44% group and native T1 > 1389ms group were significantly lower than that in ECV ≤ 44% group and native T1 ≤ 1389ms group, and it was not related to the presence of LGE. In the stepwise multivariable Cox regression model, after adjusting for age, gender, cardiac biomarkers, Mayo stage, and LGE, ECV and native T1 were independent predictors of all-cause mortality in patients with CA. Banypersad SM et al. [21] found that the increase of native T1 and ECV were associated with cardiac biomarkers and poor prognosis, and ECV > 45% was associated with decreased survival in patients with AL. Agha AM et al. [25] reported that ECV ≥ 50% was associated with increased mortality in patients with CA. In this study, ECV > 44% is related to the decreased survival rate in patients with CA, which is basically consistent with previous studies. Lin L et al. [26] demonstrated that compared with other clinical and imaging parameters, ECV ≥ 44% and LGE could independently predict the mortality in patients with CA, and in subgroups with the same LGE pattern, ECV ≥ 44% could still predict prognosis, while native T1 could not predict mortality. However, our results showed that LGE was not a predictor of death in patients with CA, and there was no significant difference in survival time between LGE-positive and LGE-negative patients, while native T1 could independently predict mortality in patients with CA.

Native T1 can be affected by different research institutions, machine types, magnetic field intensity, pulse sequence and other factors [27–29], however, ECV is based on the ratio of myocardial T1 value before and after enhancement, which can correct the influence of various technical factors mentioned above. Therefore, ECV is a relatively stable reference indicators. However, the kidney is also one of the most common organs involved in AL patients [30, 31], and renal insufficiency is a contraindication for the use of contrast media, which will limit the use of ECV in this population. Native T1 is the myocardial T1 value measured without contrast medium, therefore, it can be used as a supplementary means of ECV to evaluate the prognosis of patients with renal insufficiency.

However, this study has some limitations. Firstly, this study is a single-center study, which limits the generality of the results and increases the possibility of selection bias. Secondly, the study has a small sample size and a low incidence of end-point events, the sample size needs to be further expanded to verify the results of the study. Thirdly, the number of patients in the LGE-positive group (76%) is much higher than that in the LGE-negative group, which may bias our results. Finally, only all-cause mortality was used to observe end events.

## Conclusion

This study supports the importance of CMR in addition to cardiac biomarkers in predicting outcomes among patients at risk of having AL CA. Our results demonstrate that native T1 and ECV are independent predictors of mortality in patients with CA, and native T1 can be used to predict the prognosis of patients with renal insufficiency.

## Data Availability

The datasets used and/or analysed during the current study available from the corresponding author on reasonable request.
